# Change the preprocedural fasting policy for contrast-enhanced CT: results of 127,200 cases

**DOI:** 10.1186/s13244-022-01173-z

**Published:** 2022-02-24

**Authors:** Heng Liu, Li Zhao, Junling Liu, Fang Lan, Li Cai, Jingqin Fang, Xue Li

**Affiliations:** 1 Present Address: Department of Radiology, Daping Hospital, Army Medical University, No. 10 Changjiang Road, Yuzhong District, Chongqing, 400042 China; 2 Department of Radiology, PLA Rocket Force Characteristic Medical Center, No. 16 Xinjiekou Outer Street, Beijing, 100088 China

**Keywords:** Tomography (X-ray computed), Contrast media, Fasting, Drug-related side effects and adverse reactions, Emetics

## Abstract

**Objectives:**

To analyze the relationship between the dietary preparation status prior to contrast-enhanced CT (CECT) and adverse drug reactions (ADR) and emetic complications.

**Methods:**

Non-emergency adult patients who underwent routine CECT in our hospital from January 2019 to December 2020 were retrospectively analyzed. Stratified dietary preparation regimens were implemented for different clinical scenarios. The relationship between actual dietary preparation status and ADR and emetic complications was analyzed.

**Results:**

A total of 127,200 cases were enrolled, including 49,676 cases in the fasting group (57 years ± 13, 56.79% men) and 77,524 cases in the non-fasting group (60 years ± 13, 54.55% men). No statistical difference was found in the overall incidence of ADR (0.211% vs. 0.254%, *p* = 0.126) or emetic complications (0.030% vs. 0.046%, *p* = 0.158) between the two groups, and no aspiration pneumonia or death occurred. For patients with an ICM-ADR history, the ADR incidence in non-fasting group was significantly lower than fasting group (2.424% vs. 12.371%, *p* = 0.002). For patients with hypertension, injection dose ≥ 100 mL, injection rate ≥ 5 mL/s, and Iopromide 370 usage, non-fasting was associated with higher ADR incidence (*p* < 0.05). 36.67% of the patients experienced unnecessary excessive fasting in practice. Excessive fasting (≥ 10 h) and more water ingestion (≥ 500 mL) within 1 h prior to CECT were associated with higher ADR incidence (*p* < 0.05).

**Conclusion:**

Unrestricted food ingestion would not increase the overall risk of ADR and emetic complications. For some special patient subgroups, non-fasting, excessive fasting, and more water ingestion were associated with higher ADR incidence.

**Supplementary Information:**

The online version contains supplementary material available at 10.1186/s13244-022-01173-z.

## Key points

- For some special patient subgroups (e.g., hypertension, injection dose ≥ 100 mL, injection rate ≥ 5 mL/s, and Iopromide 370 usage), non-fasting was associated with higher ADR incidence.
- For patients with an ICM-ADR history, non-fasting was associated with significantly reduced ADR incidence.
- Excessive fasting (≥ 10 h) and more water ingestion (≥ 500 mL) within 1 h prior to CECT were associated with higher ADR incidence.

## Introduction

As a time-honored tradition, preprocedural fasting for 4–6 h is typically required prior to contrast-enhanced CT (CECT) in most medical institutions worldwide, to reduce the possibility of adverse consequences (e.g., emetic complications and aspiration) after injection of iodinated contrast media (ICM) [1–3]. Although this policy lacks methodologically acceptable evidence, and the fasting time durations and contents vary considerably in different medical institutions, preprocedural fasting is still a fairly common request worldwide [3]. Nowadays, with the extensive applications of non-ionic ICM, the incidence of emetic complications has reduced to an extremely low level (nausea 0.013%, vomiting 0.059%) [4], which is much lower than that reported by Katayama et al. about 30 years ago [5]. According to our rigorous and extensive literature review [3], no aspiration pneumonia has been reported due to the lenient implementation of preparatory fasting. Non-fasting would not increase the risk of aspiration pneumonia and the incidence of emetic complications [3]. Furthermore, preprocedural fasting may bring negative effects, including but not limited to general discomfort (e.g., hunger, thirst, dehydration, anxiety, irritability), hypoglycemic risk in diabetic patients, and may even increase the risk of adverse events [3]. Therefore, it seems not prudent to fast without distinction for every patient without evidence-based considerations, and the existing fasting strategies can reasonably be less restrictive.

Considering the possible negative effects of fasting and potential benefits of abolishing fasting orders, the latest European Society of Urogenital Radiology (ESUR) guidelines and American Committee of Radiology (ACR) guidelines clarified that fasting was not recommended prior to routine intravenous ICM injection [6, 7]. These updates officially provide a framework and guidelines for radiological nursing practices prior to CECT. Unfortunately, the guidelines did not give specific recommendations on the types of allowed food, specific clinical scenarios, and special patient subgroups that require additional attention. The decision-makers still face embarrassment and challenge due to unavailable standards, and decisions often rely on deep-rooted clinical experience. At present, the sample sizes of only a few researches were quite small [8–11], and the majority of them focused on a single patient population [8, 9, 12]. No hierarchical management of dietary preparation regimens for specific clinical scenarios was explored. The relationship between whether fasting or not and the overall incidence of ADR and emetic complications was roughly analyzed, and no in-depth detailed comparisons was performed between abdominal and non-abdominal examinations, inpatients and outpatients, different fasting time durations, and different amounts of water ingestion.

From January 2019, following the dietary policy in the latest ICM guidelines, our hospital undertook a quality improvement program involving a stratified dietary preparation regimen for different clinical scenarios. The purpose of this large-scale study was to analyze the relationship between the dietary preparation status and ADR, and emetic complications, and to provide solid data support for the supplementation and standardized promotion of ICM guidelines.

## Materials and methods

### Study participants

This study was approved by the institutional review board of our hospital (ratification number: 2021(146)). Due to its retrospective nature, the written informed consent was exempted. The data of non-emergency adult patients who underwent routine CECT in our hospital from January 2019 to December 2020 were analyzed. Inclusion criteria: (i) Patients who met the indications for enhanced CT examination [6, 7]; (ii) Patients equal to or older than 18 years and received routine enhanced CT examination; (iii) Patients with complete clinical ADR data records. Exclusion criteria: (i) Patients younger than 18 years; (ii) Emergency patients; (iii) Patients with incomplete form data filling.

### Stratified dietary preparation regimen

The American Society of Anesthesiology guidelines [13], multidisciplinary consensus by International Committee for the Advancement of Procedural Sedation [14], CT examination technology expert consensus [15], preoperative fasting literature [16], the gastrointestinal endoscopy guidelines [17], and the literature on dietary preparation prior to CECT [8–11] were referred. Following the dietary policy in the latest ICM guidelines, a stratified dietary preparation regimen was implemented for different clinical scenarios (Table 1). The dietary preparation instructions were notified by the radiology nurses when the clinicians made the appointment. The radiology nurses provided health education to patients or their family members and explained the importance of following the dietary preparation policy.

**Table 1:** Preparative dietary protocols at our institution

	Dietary preparation instructions	Solid food	Low residue/fiber food	Clear liquids
Clinical scenarios		Rice, steamed buns, noodles, bread, meat, vegetables, fruits, eggs, etc.	Congee, yogurt, milk, soy milk, soup, etc.	Water, juice, non-carbonated beverages, tea, etc.
Abdominal examination	The upper abdomen, the whole abdomen, or the combined examination involving the upper abdomen or the whole abdomen^1^ (the detection of the gastrointestinal cavity lesions is excluded)	Fast for 4 h	Fast for 3 h	Unrestricted ingestion
	The lower abdomen (kidneys, adrenal glands, renal artery, urinary system, etc.), pelvic cavity (bladder, uterus, appendages, prostate, etc.)	Unrestricted ingestion (greasy and fried foods are excluded)	Unrestricted ingestion	Unrestricted ingestion
	Three-dimensional imaging of the lumen (such as gastrointestinal cavity enhanced CT)	Fast for 4–6 h	Fast for 3 h	Unrestricted ingestion
Non-abdominal examination	Contrast-enhanced CT and angiography of head, neck, chest, limbs and other parts	Unrestricted ingestion (greasy and fried foods are excluded)	Unrestricted ingestion	Unrestricted ingestion
Special population	Severe esophageal diseases or gastric emptying disorders, patients at risk of aspiration^2^, patients require fasting for clinical treatment, examination under general anesthesia or sedation	Foods and water were restricted according to clinical treatment needs. Intravenous supplementation of sugar and salt water was performed when necessary		

^1^The dietary preparation regimen of patients undergoing multi-site combined examination was based on the site that required fasting

^2^The risk of aspiration included disturbance of consciousness, increased intracranial pressure, and impaired swallowing reflex, and so on

### ICM used and injection protocol

GE LightSpeed VCT^®^ (GE Healthcare, Milwaukee, WI) and Philips Brilliance iCT Scanner (Royal Dutch Philips Electronics Ltd, Amsterdam, The Netherlands) were used for CT scanning. Nonionic iodinated contrast media were intravenously injected by a high-pressure injector (Ulrich Medical^®^ Inc.). The ICM used included Iopromide 370 (Bayer Healthcare), Iodixanol 270 (GE Healthcare), Ioversol 320 (Jiangsu Hengrui Medicine Co., Ltd), Iohexol 350 (Yangtze River Pharmaceutical Co., Ltd), Iopamidol 350 (Bracco), Iobitridol 350 (Guerbet), and Iodixanol 320(Jiangsu Hengrui Medicine Co., Ltd). The injection doses and rates of ICM were selected according to patient weight and the purpose of CT examinations, based on our institutional protocol (Additional file 1: Table S1).

### Evaluation content and quality control

The radiology nurses recorded the patients' actual dietary preparation prior to the examination, including the fasting contents, fasting duration, and the amount of water ingestion within 1 h prior to the examination. Excessive fasting was defined as a fasting duration ≥ 10 h. More water ingestion was defined as a water ingestion ≥ 500 mL within 1 h prior to the examination. All patients signed an ICM usage informed consent form prior to the examination. The radiology nurses filled out the ICM usage evaluation form, mainly including basic information of patients, risk factors and underlying diseases, examination sites, ICM names, injection doses and injection rates. For patients who developed ADR, the radiology nurses filled out the ADR record form, and recorded the occurrence, treatment, and outcome of ADR and emetic complications in detail. Thyroid toxicity and post-contrast acute kidney injury were not follow-up observed in this study. The severity of ADR was determined according to the 2021 ACR guidelines [7]. The radiologists evaluated the occurrence of aspiration pneumonia within 4 days through the feedback of respiratory symptoms and subsequent chest CT imaging [9, 18]. Standardized electronic documents were used to input and save data. Two radiology nurses with over 8 years of work experience checked the original data blind-to-blind to ensure the data accuracy and completeness.

### Statistical methods

Continuous variables were described by mean values and standard deviation. Counting data was presented in terms of frequencies and percentages (%). Chi-square test was performed on SPSS 22.0 (IBM, Chicago, USA), and Pearson Chi-square test or Fisher's exact test was used for rates comparison. *p* < 0.05 was considered statistically significant.

## Results

### Demographic and baseline characteristics

**Fig. 1:**
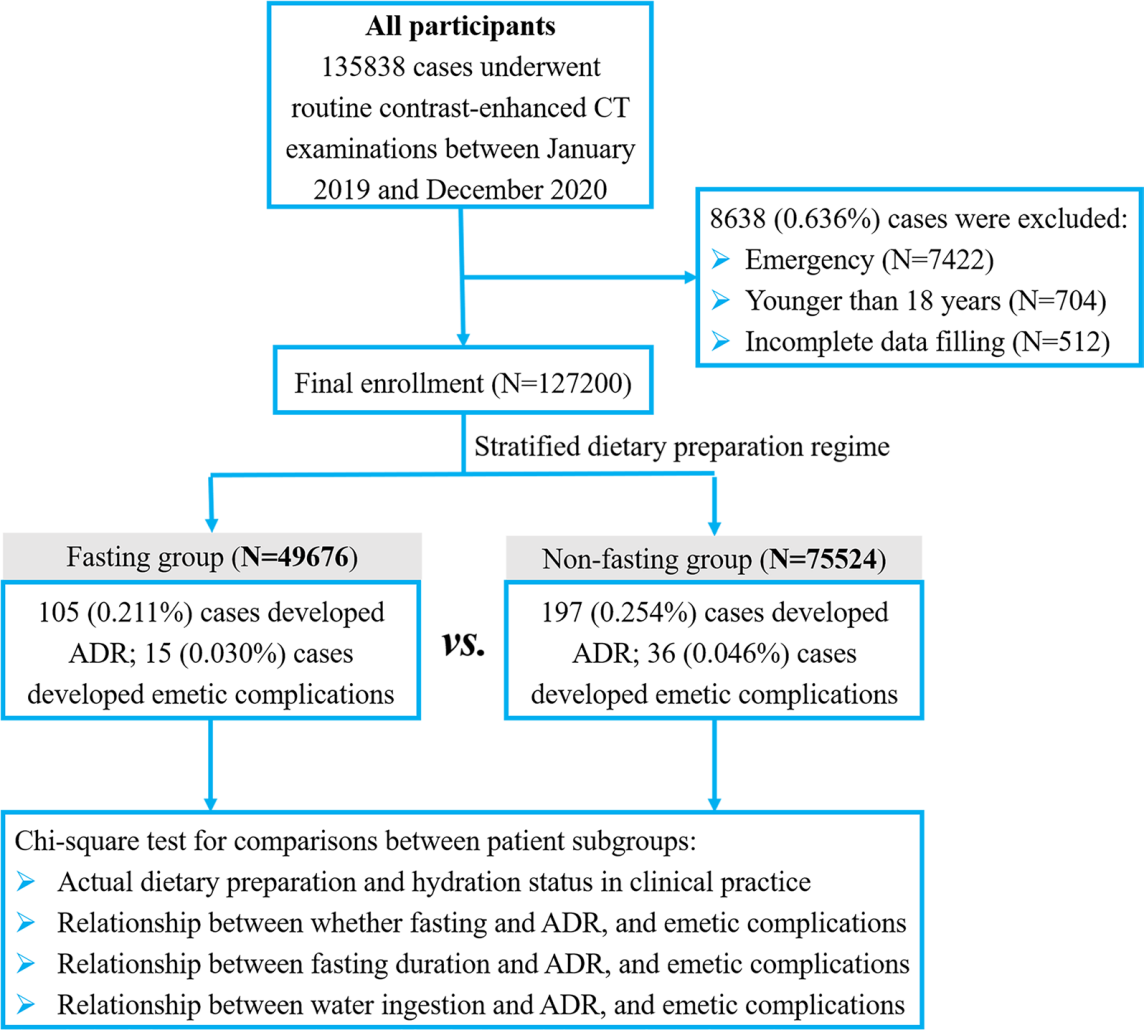
Flowchart illustrating population enrollment

The baseline data of patients are shown in Table 2. During the observation period, a total of 135,838 cases underwent routine enhanced CT examinations. Among them, 8638 cases were excluded, including 7422 emergency patients, 704 patients younger than 18 years, and 512 patients with incomplete form data filling. As a result, 127,200 eligible cases were enrolled in the analysis (Fig. 1), including 49,676 cases (57 years ± 13, 28,210 men [56.79%]) in the fasting group, and 77,524 cases (60 years ± 13, 42,287 men [54.55%]) in the non-fasting group.

**Table 2:** Summary of patient characteristics

	Fasting group (%)	Non-fasting group (%)
Total number of patients	49,676 (39.05)	77,524 (60.95)
Gender		
Male	28,210 (56.79)	42,287 (54.55)
Female	21,466 (43.21)	35,237 (45.45)
Age (years)		
Mean ± standard deviation	57 ± 13	60 ± 13
< 70	40,302 (81.13)	60,721 (78.33)
≥ 70	9374 (18.87)	16,803 (21.67)
Patient source		
Inpatients	21,106 (42.49)	40,173 (51.82)
Outpatients	28,570 (57.51)	37,351 (48.18)
Examination sites		
Abdominal	30,462 (61.32)	15,396 (19.86)
Non-abdominal	19,214 (38.68)	62,128 (80.14)
Risk factors and underlying diseases		
ICM-ADR history	97 (0.20)	165 (0.21)
Other ADR histories	3536 (7.12)	6738 (8.69)
Asthma	110 (0.22)	194 (0.25)
Hypertension	11,411 (22.97)	22,718 (29.30)
Coronary heart disease	1608 (3.24)	4668 (6.02)
Heart failure	173 (0.35)	323 (0.42)
Renal insufficiency	99 (0.20)	151 (0.19)
Hyperthyroidism	16 (0.03)	45 (0.06)
Tumor radio-chemotherapy	5391 (10.85)	5324 (6.87)
β blockers	1151 (2.32)	12,965 (16.72)
Diabetes	2079 (4.19)	3945 (5.09)
ICM name		
Iopromide 370	7833 (15.77)	12,532 (16.17)
Iodixanol 270	3936 (7.92)	5490 (7.08)
Ioversol 320	6693 (13.47)	12,244 (15.79)
Iohexol 350	17,015 (34.25)	26,764 (34.52)
Iopamidol 350	5603 (11.25)	9625 (12.42)
Iobitridol 350	2993 (6.03)	4392 (5.67)
Iodixanol 320	5603 (11.28)	6477 (8.35)
Injection dose		
< 100 mL	46,817 (94.24)	72,579 (93.62)
≥ 100 mL	2859 (5.76)	4945 (6.38)
Injection rate		
< 5 mL/s	36,836 (74.15)	49,524 (63.88)
≥ 5 mL/s	12,840 (25.85)	28,000 (36.12)

The same patient might have multiple risk factors and underlying diseases simultaneously

*ICM* iodinated contrast media, *ADR* adverse drug reactions

### Incidence of ADR and emetic complications

**Fig. 2:**
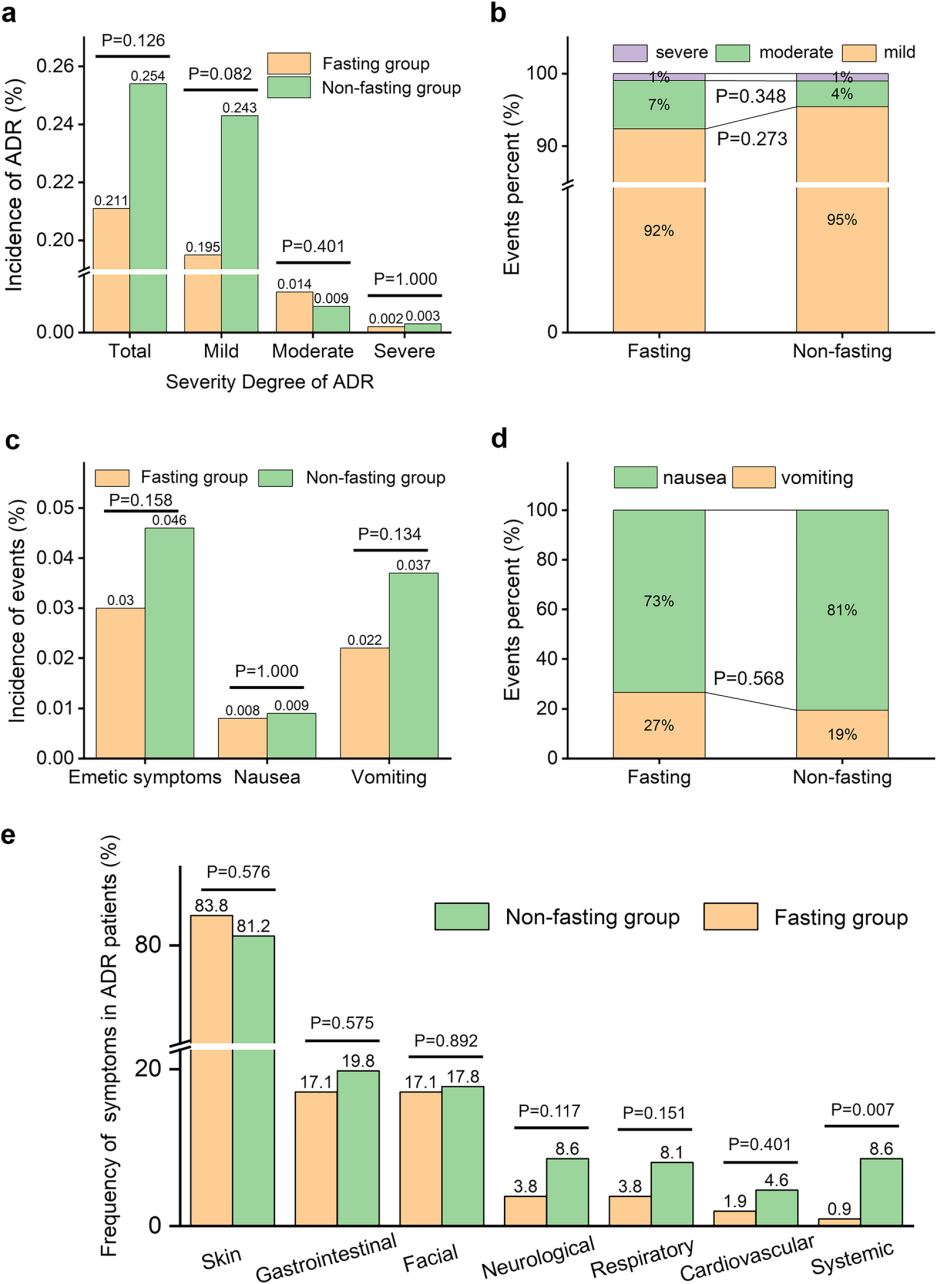
**a** The ADR incidence. **b** The proportion of ADR with different severity degrees in patients who developed ADR. **c** The incidence of emetic complications. **d** The proportion of nausea and vomiting in patients who developed emetic complications. **e** The frequency of various symptoms in ADR patients

The overall ADR incidence was 0.237% (302/127,200), and the overall incidence of emetic complications was 0.041% (51/127,200), and no aspiration pneumonia or death occurred. No statistical difference was found in the overall ADR incidence, the incidence of ADR with different severity degrees, and their proportion between the fasting group and the non-fasting group (*p* > 0.05, Fig. 2a, b). No statistical difference was found in the incidence of emetic complications, nausea and vomiting, and their proportion between the two groups (*p* > 0.05, Fig. 2c, d). Whether fasting or not, the most frequent symptoms in patients who developed ADR were skin symptoms, followed by gastrointestinal symptoms and facial symptoms (Fig. 2e). The ADR occurrence of patients in different subgroups is shown in Table 3. For patients receiving abdominal examinations, the ADR incidence in the non-fasting group was lower than that in the fasting group (0.117% vs. 0.309%, *p* < 0.001). For patients receiving non-abdominal examinations, the ADR incidence in the non-fasting group was higher than that in the fasting group (0.288% vs. 0.057%, *p* < 0.001). Among various risk factors, patients with an ICM-ADR history showed the highest overall ADR incidence (6.11%, 16/262), and the ADR incidence in the non-fasting group was remarkably lower than that in the fasting group (2.424% vs. 12.371%, *p* = 0.001). For patients with hypertension, injection dose ≥ 100 mL, injection rate ≥ 5 mL/s, and Iopromide 370 usage, the ADR incidence in the non-fasting group was higher than that in the fasting group (*p* = 0.037, < 0.001, < 0.001, 0.014, respectively).

**Table 3:** The ADR occurrence of patients in different patient subgroups

	Fasting group (%)	Non-fasting group (%)	*P* value
Total number of patients	105/49,676 (0.211)	197/77,524 (0.254)	0.126
Examination sites			
Abdominal	94/30,462 (0.309)	18/15,396 (0.117)	< 0.001
Non-abdominal	11/19,214 (0.057)	179/62,128 (0.288)	< 0.001
Patient source			
Inpatients	44/21,106 (0.208)	93/40,173 (0.231)	0.566
Outpatients	61/28,570 (0.214)	104/37,351 (0.278)	0.098
Risk factors and underlying diseases			
ICM-ADR history	12/97 (12.371)	4/165 (2.424)	0.001
Other ADR histories	11/3536 (0.311)	24/6738 (0.341)	0.709
Asthma	0/110	2/194 (1.031)	N/A
Hypertension	16/11,411 (0.140)	57/22,718 (0.251)	0.037
Coronary heart disease	1/1608 (0.062)	12/4668 (0.257)	0.244
Heart failure	0/173	2/323 (0.619)	N/A
Renal insufficiency	1/99 (1.010)	0/151	N/A
Hyperthyroidism	0/16	0/45	N/A
Tumor radio-chemotherapy	10/5391 (0.185)	7/5324 (0.131)	0.482
β blockers	1/1151 (0.087)	41/12,965 (0.316)	0.277
Diabetes	0/2079	13/3945 (0.330)	N/A
Age ≥ 70 years	11/9374 (0.117)	20/16,803 (0.119)	0.970
Injection dose ≥ 100 mL	2/22,859 (0.070)	25/4945 (0.506)	< 0.001
Injection rate ≥ 5 mL/s	2/12,840 (0.016)	96/28,000 (0.343)	< 0.001
ICM name			
Iopromide 370	22/7833 (0.218)	64/12,532 (0.511)	0.014
Iodixanol 270	12/3936 (0.305)	25/5490 (0.455)	0.249
Ioversol 320	7/6693 (0.105)	21/12,244 (0.172)	0.252
Iohexol 350	15/17,015 (0.088)	27/26,764 (0.101)	0.675
Iopamidol 350	14/5603 (0.250)	14/9625 (0.145)	0.147
Iobitridol 350	11/2993 (0.368)	10/4392 (0.228)	0.268
Iodixanol 320	24/5603 (0.428)	36/6477 (0.556)	0.320

The same patient might have multiple risk factors and underlying diseases simultaneously. N/A, unapplicable for statistics due to the sample size

*ICM* iodinated contrast media, *ADR* adverse drug reactions

### The relationship between actual dietary preparation and the incidence of ADR and emetic complications

**Fig. 3:**
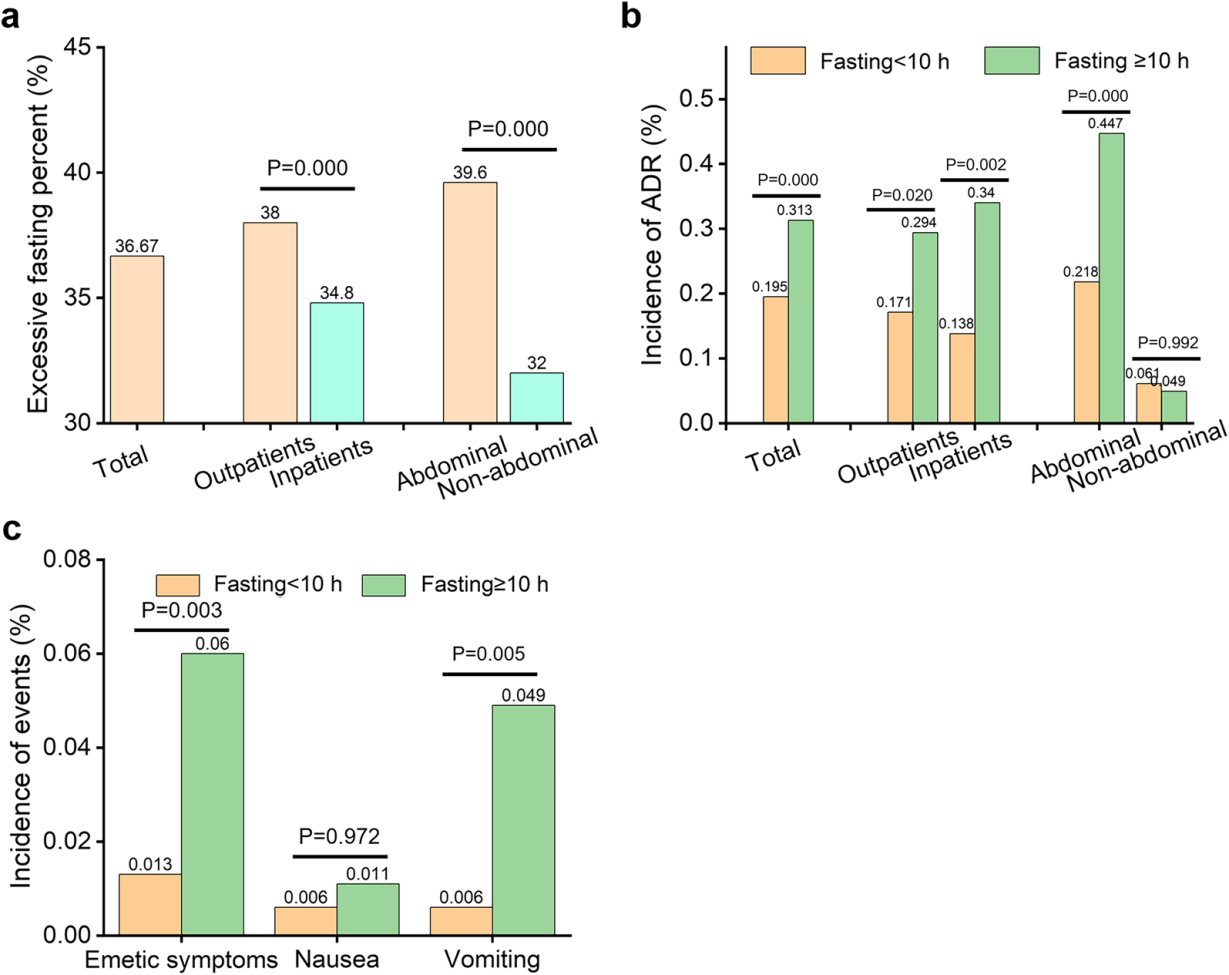
**a** Analysis of actual excessive fasting profile. **b** The ADR incidence in patients with different fasting durations. **c** The incidence of emetic complications in patients with different fasting durations

The actual excessive fasting profile is shown in Fig. 3a. The mean fasting duration in the fasting group was 9.0 ± 5.3 h, and 36.67% (18,214/49,676) of the patients experienced excessive fasting (≥ 10 h). The proportion of excessive fasting in outpatients was greater than that in inpatients (38.0% vs. 34.8%, *p* < 0.001). The proportion of excessive fasting in abdominal examination patients was greater than that in non-abdominal examination patients (39.6% vs. 32.0%, *p* < 0.001). The ADR incidence in excessive fasting patients was higher than that in patients with fasting < 10 h (0.313% vs. 0.195%, *p* < 0.001, Fig. 3b). For inpatients, outpatients, and abdominal examination patients, the ADR incidence in excessive fasting patients was higher than that in patients with fasting < 10 h (*p* = 0.002, 0.020, < 0.001, respectively). The incidence of emetic complications and vomiting in excessive fasting patients was higher than that in patients with fasting < 10 h (*p* = 0.003, 0.005, respectively, Fig. 3c). There was no correlation between the nausea incidence and fasting duration (*p* = 0.972).

**Fig. 4:**
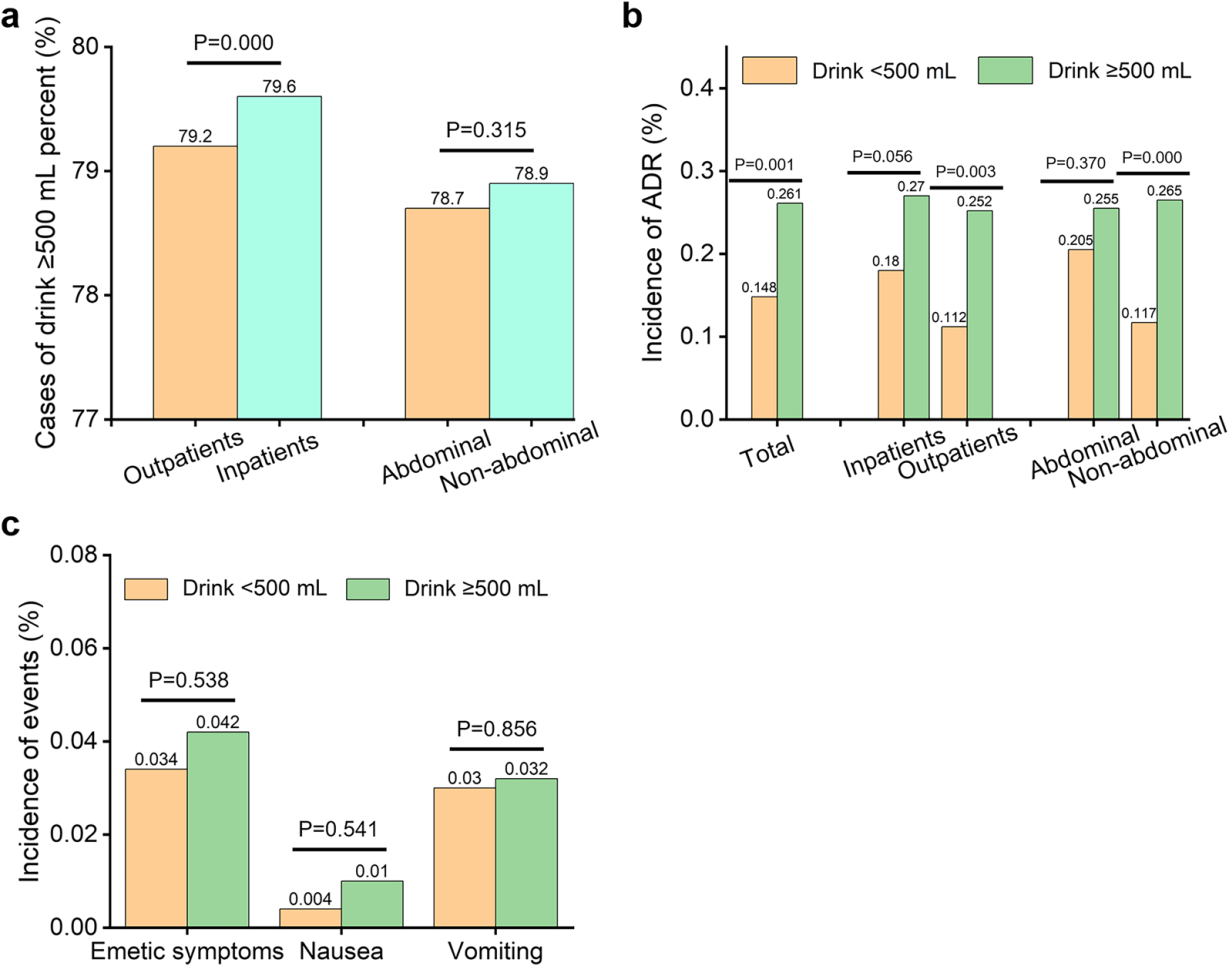
**a** Analysis of actual water ingestion within 1 h prior to the examination. **b** The ADR incidence in patients with different amounts of water ingestion. **c** The incidence of emetic complications in patients with different amounts of water ingestion

The proportion of patients with more water ingestion (≥ 500 mL) within 1 h prior to the examination in inpatients was higher than that in outpatients (79.6% vs. 79.2%, *p* < 0.001, Fig. 4a). The ADR incidence in patients with more water ingestion was higher than that in patients with water ingestion < 500 mL (0.261% vs. 0.148%, *p* = 0.001, Fig. 4b). For inpatients and non-abdominal examination patients, the ADR incidence in patients with more water ingestion was higher than that in patients with water ingestion < 500 mL (*p* = 0.003, < 0.001, respectively). There was no correlation between the amount of water ingestion and the incidence of emetic complications, nausea, and vomiting (*p* > 0.05, Fig. 4c).

## Discussion

### Incidence of ADR and emetic complications

The overall incidence of ADR and emetic complications was lower than that in previous literature. This may be attributed to the whole-process standardized management of patients before, during, and after the examination in our institution [4, 19, 20]. There was no significant difference in the incidence and proportion of different severity degrees of ADR, the incidence of emetic complications, and the proportion of nausea and vomiting between the two groups (*p* > 0.05). It indicated that fasting would not increase the overall risk of adverse events, consistent with the literature [4, 8, 21]. The frequency of systemic symptoms in ADR patients in the non-fasting group was about 10 times higher than that in the fasting group (*p* = 0.007). It suggests that more adequate emergency preparedness against systemic symptoms should be ready for patients without fasting.

Some abdominal examination patients suffered from gastrointestinal diseases and were prone to emetic complications. When examining the gastrointestinal tract and adjacent lesions, patients were recommended to fast to avoid the interfere of the gastrointestinal contents on the image quality. Based on these considerations, patients were classified into abdominal examination patients and non-abdominal examination patients. Surprisingly, regardless of examination sites, the ADR incidence was significantly different (*p* < 0.001) between the fasting group and the non-fasting group, while the ADR levels were opposite. This might lead to an illusion that there was no statistical difference in the overall ADR incidence between the two groups (*p* = 0.126). In some previous studies, a "false negative" conclusion that dietary preparation status was not associated with the ADR occurrence was hastily obtained after a preliminary comparison of the overall ADR incidence between the two groups. However, no further in-depth subgroup analysis on the relationship between dietary preparation status and ADR occurrence in different subgroups was performed.

Among the various risk factors, patients with an ICM-ADR history showed the highest overall ADR incidence, consistent with previous literature [4]. The ADR incidence in the non-fasting group was about 80% lower than that in the fasting group, which was equivalent to eliminating the risk of ADR recurrence in 4 out of every 5 high-risk patients. Considering that repeated ADR in patients with an ICM-ADR history is one of the greatest challenges in CECT [22–24], such a remarkable effect suggests that unrestricted food ingestion may be an awesomely simple and effective approach to reduce such risks. For patients with hypertension, injection dose ≥ 100 mL, injection rate ≥ 5 mL/s, and Iopromide 370 usage, the ADR incidence in the non-fasting group was higher than that in the fasting group (*p* < 0.05). Although the mechanism is unclear, it suggests that these risk factors should be paid close attention when implementing the dietary policy in the latest ICM guidelines.

### The relationship between actual dietary preparation status and the incidence of ADR and emetic complications

Although the latest ICM guidelines clearly stated that there was no need to fast prior to routine ICM injection [6, 7], and this policy had been fully explained to patients, 36.67% of patients still experienced unnecessary excessive fasting in clinical practice. The explanation may be that the majority of clinicians lack an in-depth understanding of the latest guidelines and policies. Based on deep-rooted clinical experience patterns, they are refractory to break the traditional fasting rules. And patients are more willing to comply with the fasting instructions provided by clinicians rather than the dietary preparation policy recommended by the radiology department. Some patients are scheduled to undergo multiple laboratory and imaging examinations on the same day, so they maintain a radical attitude toward fasting. The results showed that the proportion of excessive fasting in abdominal examination patients was higher than that in non-abdominal examination patients (*p* < 0.001). The reason may be that a considerable proportion of abdominal examination patients suffer from gastrointestinal diseases. Considering the clinical treatment needs, and worry that eating will interfere with the image quality of the gastrointestinal and adjacent lesions, clinicians often encourage patients to keep fasting until finishing the CT examination. The proportion of excessive fasting in inpatients was lower than that in outpatients (*p* < 0.001). This may be related to the support of the stratified dietary preparation regimen, timely reminders, and encouragement to the inpatients by some clinicians.

The incidence of ADR, emetic complications, and vomiting in excessive fasting patients were higher than that of patients with fasting for less than 10 h (*p* < 0.01), indicating that excessive fasting would increase the incidence of ADR and emetic complications, especially vomiting symptoms, consistent with the literature [25, 26]. The reason may be that excessive fasting will increase the patients' stress response to enter into a catabolic state. This would disrupt the body's internal environment and metabolic balance and increase the risk of adverse events after ICM injection [27, 28]. Patients were prone to irritability, anxiety, poor compliance, and non-cooperation during waiting and examination [10]. Elderly patients with malnutrition and weak constitution are often more prone to develop physical discomfort. The stratified dietary preparation regimen could ensure the patients in normal metabolic states, improve their comfort and cooperation, reduce the risk of adverse events for special subgroups, and avoid unnecessary delay or cancellation of examinations. This is of great significance for improving the clinical benefits of patients and the quality of radiology nursing practices.

In clinical practice, it has become common practice to strongly encourage patients to replenish fluids intravenously or orally before and after the examination, to prevent possible contrast-induced nephropathy in high-risk patients [6, 7, 29]. Within 1 h prior to the examination, the proportion of more water ingestion in inpatients was higher than that in outpatients (*p* < 0.001), indicating that inpatients had a better executive ability of oral hydration. This may be attributed to the consistent understanding of the importance of hydration, timely reminders and encouragement to inpatients by clinicians. Although the ICM guidelines have always emphasized the importance of hydration [6, 7, 30], there is no literature exploring the relationship between water ingestion in a short time and ADR occurrence yet. Our results showed for the first time that for inpatients and non-abdominal examination patients, more water ingestion was associated with higher ADR incidence (*p* < 0.01). This suggested that oral hydration prior to the examination should be carried out in a more continuous and gentle manner, avoiding consuming large amounts of fluids in a very short time. The exact mechanism needs further investigations.

This study has limitations. Firstly, although standardized training guaranteed the consistency of the operation and evaluation standards, the confounding factors were not adjusted when comparison between groups. As the sample size was large enough, we believed that the confounding factors were evenly distributed between groups. Secondly, in practice, we found a significant reduction in the number of patients who delayed the examination due to unsatisfactory dietary preparation, and a significant reduction in the feedback from patients on adverse events such as thirst, hunger, dehydration, and hypoglycemia. However, the exact number of these cases was not recorded in detail. Thirdly, this study observed an association between dietary preparation status and the occurrence of adverse events in some patient subgroups. Prospective multicenter randomized controlled trials are needed to further clarify whether there is a causal relationship.

## Conclusion

Unrestricted food ingestion would not increase the overall incidence of ADR and emetic complications, and the risk of aspiration pneumonia. In practice, a considerable proportion of patients experienced unnecessary excessive fasting. Excessive fasting and more water ingestion within 1 h prior to the examination were associated with ADR occurrence. For special patient subgroups, it is necessary to comprehensively weigh by combining specific clinical situations, formulate personalized dietary preparation plans, and emphasize the importance of following the dietary preparation principles.

## Supplementary Information

**Additional file 1.** For the injection protocol of ICM and dietary preparation principles in our practice, and supplementary methods, results and discussion.

## Data Availability

All data generated or analyzed during this study are included in this published article and its Additional information files.

## Abbreviations

ACR: American Committee of Radiology
ADR: Adverse drug reactions
CECT: Contrast-enhanced CT
ESUR: European Society of Urogenital Radiology
ICM: Iodinated contrast media
