# Supplementary material for: Change the preprocedural fasting policy for contrast-enhanced CT: results of 127,200 cases
Source: Insights Imaging. 2022 Feb 24;13:29. doi: 10.1186/s13244-022-01173-z (PMC8873329; doi:10.1186/s13244-022-01173-z)
Supplement: Supplementary file 1 — Additional file 1. For the injection protocol of ICM and dietary preparation principles in our practice, and supplementary methods, results and discussion. [file 13244_2022_1173_MOESM1_ESM.docx]

**ELECTRONIC SUPPLEMENTARY MATERIAL**

**Supplementary Table 1.** The injection protocol of ICM in our practice

|  | Iodine content in ICM used (mg I/mL) | Head and neck CECT | Chest CECT | Abdominal and pelvic CECT | Head and neck CTA | Coronary CTA | Body CTA |
| --- | --- | --- | --- | --- | --- | --- | --- |
| Injection dose (mL/kg body weight) | 270 | 1.1 | 1.1 | 1.4 | 1.1 | 1.1 | 1.4 |
|  | 320 | 1.0 | 1.0 | 1.3 | 1.1 | 1.0 | 1.3 |
|  | 350 | 0.9 | 0.9 | 1.2 | 1.1 | 0.9 | 1.2 |
|  | 370 | 0.8 | 0.8 | 1.1 | 1.1 | 0.8 | 1.1 |
| Injection rate (mL/s) | 270 | 3-5 | 3-5 | 3-5 | 4-6.5 | 4-6.5 | 4-6.5 |
|  | 320 | 2.5-5 | 2.5-5 | 2.5-5 | 4-6.5 | 4-6.5 | 4-6.5 |
|  | 350 | 2.5-5 | 2.5-5 | 2.5-5 | 4-6.5 | 3.5-6.5 | 3.5-6.5 |
|  | 370 | 2.5-5 | 2.5-5 | 2.5-5 | 3.5-6.5 | 3.5-6.5 | 3.5-6.5 |

The average injection dose and injection rate was 65 ± 16 mL and 4.0± 1.1 mL/s, respectively.

For patients with a history of ICM-ADR, dietary preparation was carried out according to the examination site requirements. For patients who did not require fasting, they were advised to conservatively consume a small amount of light meal to maintain the minimum energy needs for safety considerations, and rarely reached the level of "gastric fullness". For patients who needed dietary preparation, the fasting time was controlled to avoid unnecessary excessive fasting. Emphasize the importance of continuous hydration and avoid drinking large amounts of water in a short period of time. For patients with a history of mild ICM-ADR, our preference was to replace the culprit ICM responsible for previous ADR, so that another non-culprit ICM could be recommended in subsequent examination procedures. For patients with a history of moderate to severe ICM-ADR, in addition to replace the culprit ICM, promethazine hydrochloride was usually intramuscularly injected one hour prior to examination. Actually, in our institution, for patients with a history of severe ICM-ADR, they were usually strongly recommended to receive alternative imaging modalities with comparable diagnostic values, rather than CECT examinations.

**Supplementary results**

The frequency of various symptoms in patients who developed ADR is shown in **Figure 2e.** Except that the systemic symptoms were more common in the non-fasting group (8.6% vs 0.9%, P=0.007), there was no statistical difference in the frequency of all other symptoms between the two groups (P>0.05).

The mean fasting duration was 10.6 ± 5.6 hours and 12.6 ± 5.8 hours in patients who developed ADR and who developed emetic complications in the fasting group, respectively. The ADR incidence in patients with different fasting durations is shown in **Figure 3b**. No correlation was found between the ADR incidence and fasting duration in patients who underwent non-abdominal examinations (P=0.992, **Figure 3b**).

The mean amount of water ingestion within 1 hour prior to the examination in inpatients and outpatients was 570.4±119.2 mL and 562.2±225.6 mL, respectively. There was no statistical difference in the proportion of patients with more water ingestion within 1 hour prior to the examination between abdominal examination patients and non-abdominal examination patients (P=0.315, **Figure 4a**). There was no correlation between the ADR incidence and the amount of water ingestion in outpatients and abdominal examination patients (P>0.05, **Figure 4b**).

**Supplementary discussion**

There is very limited big data supporting the dietary preparation policy prior to CECT in the latest ICM guidelines. Due to the extremely low incidence of emetic complications, large sample size is crucial to guarantee scientific and reliable conclusions. As the largest clinical study (127200 cases) in this field so far, this study implemented a stratified dietary preparation regimen for different clinical scenarios on the basis of dietary policy in the latest ICM guidelines, systematically analyzed the relationship between actual dietary preparation status (e.g., whether fasting or not, fasting duration, amount of water ingestion within 1 hour prior to the examination) and the incidence of ADR and emetic complications in different population subgroups. The study is expected to provide solid data support for the supplementation and standardized promotion of ICM guidelines.
